# Resveratrol Inhibits Key Steps of Steroid Metabolism in a Human Estrogen-Receptor Positive Breast Cancer Model: Impact on Cellular Proliferation

**DOI:** 10.3389/fphar.2018.00742

**Published:** 2018-07-10

**Authors:** Stefan Poschner, Alexandra Maier-Salamon, Martin Zehl, Judith Wackerlig, Daniel Dobusch, Anastasia Meshcheryakova, Diana Mechtcheriakova, Theresia Thalhammer, Bettina Pachmann, Walter Jäger

**Affiliations:** ^1^Division of Clinical Pharmacy and Diagnostics, Department of Pharmaceutical Chemistry, University of Vienna, Vienna, Austria; ^2^Department of Analytical Chemistry, Faculty of Chemistry, University of Vienna, Vienna, Austria; ^3^Division of Drug Design and Medicinal Chemistry, Department of Pharmaceutical Chemistry, University of Vienna, Vienna, Austria; ^4^Department of Pathophysiology and Allergy Research, Center for Pathophysiology, Infectiology and Immunology, Medical University of Vienna, Vienna, Austria; ^5^Vienna Metabolomics Center, University of Vienna, Vienna, Austria

**Keywords:** resveratrol, breast cancer, MDA-MB-231, MCF-7, steroids, metabolomics

## Abstract

The role of resveratrol (RES) in preventing breast cancer is controversial, as low concentrations may stimulate the proliferation of estrogen-receptor alpha positive (ERα+) breast cancer cells. As metabolism is the key factor in altering cellular estrogens, thereby influencing breast tumor growth, we investigated the effects of RES on the formation of estrogen metabolites, namely 4-androstene-3,17-dione (AD), dehydroepiandrosterone (DHEA), dehydroepiandrosterone-3-*O*-sulfate (DHEA-S), estrone (E1), estrone-3-sulfate (E1-S), 17β-estradiol (E2), 17β-estradiol-3-*O*-(β-D-glucuronide) (E2-G), 17β-estradiol-3-*O*-sulfate (E2-S), 16α-hydroxy-17β-estradiol (estriol, E3), and testosterone (T) in ERα- MDA-MB-231 and ERα+ MCF-7 cells. Incubation of both of the cell lines with the hormone precursors DHEA and E1 revealed that sulfation and glucuronidation were preferred metabolic pathways for DHEA, E1 and E2 in MCF-7 cells, compared with in MDA-MB-231 cells, as the V_max_ values were significantly higher (DHEA-S: 2873.0 ± 327.4 fmol/10^6^ cells/h, E1-S: 30.4 ± 2.5 fmol/10^6^ cells/h, E2-S: 24.7 ± 4.9 fmol/10^6^ cells/h, E2-G: 7.29 ± 1.36 fmol/10^6^ cells/h). RES therefore significantly inhibited DHEA-S, E1-S, E2-S and E2-G formation in MCF-7, but not in MDA-MB-231 cells (K_i_s: E2-S, 0.73 ± 0.07 μM < E1-S, 0.94 ± 0.03 μM < E2-G, 7.92 ± 0.24 μM < DHEA-S, 13.2 ± 0.2 μM). Suppression of these metabolites subsequently revealed twofold higher levels of active E2, concomitant with an almost twofold increase in MCF-7 cell proliferation, which was the most pronounced upon the addition of 5 μM RES. As the content of RES in food is relatively low, an increased risk of breast cancer progression in women is likely to only be observed following the continuous consumption of high-dose RES supplements. Further long-term human studies simultaneously monitoring free estrogens and their conjugates are therefore highly warranted to evaluate the efficacy and safety of RES supplementation, particularly in patients diagnosed with ERα+ breast cancer.

## Introduction

Breast cancer is a major cause of death in women worldwide (Ferlay et al., 2015). Chemoprevention is therefore crucial to reduce morbidity and mortality. Several epidemiological and experimental studies have indicated that certain natural phenolic compounds may inhibit mammary carcinogenesis, and may therefore act as chemopreventive agents (Pan et al., 2015). One of such compound is *trans*-resveratrol (3,5,4′-trihydroxy-*trans*-stilbene), a naturally occurring polyphenol found in red wine and foods, including peanuts, blueberries and cranberries, as well as the skin of grapes (Aiyer et al., 2012). The anti-proliferative properties of RES have been demonstrated *in vitro* against hormone-dependent and hormone-independent breast cancer cells through the induction of apoptosis via the down-regulation of p53, NF-κB and Bcl-2, the inhibition of ribonucleotide reductase and DNA polymerases, and the suppression of the RhoA/Lats1/YAP signaling axis (Saluzzo et al., 2016; Kim et al., 2017). RES is also a radical scavenger and an inhibitor of cyclooxygenases (COX-1 and COX-2), which partly explains why this compound may reduce the occurrence of breast cancer (Murias et al., 2005). In addition, animal experiments identified significantly reduced tumor growth in human breast cancer xenografts subsequent to RES treatment, therefore supporting the use of this polyphenol as a chemotherapeutic agent (Garvin et al., 2006). Furthermore, a human case-control study using data from 369 cases and 602 controls reported a significant inverse association for the relationship between dietary RES intake and the risk of developing breast cancer (Levi et al., 2005).

However, based on the estrogen-like effects of RES due to its structural similarity with 17β-estradiol (E2) (Yildiz, 2005), some researchers and clinicians are concerned that the intake of RES may negatively affect hormone-dependent malignancies. Indeed, RES stimulates the proliferation of estrogen-receptor alpha positive (ERα+) breast cancer cells at low concentrations, but inhibits tumor growth at high doses. In ER alpha negative (ERα-) cells, this biphasic effect has not been observed; RES only exhibits anti-proliferative effects (Basly et al., 2000).

In addition to interactions with ERs, the stimulatory effects of RES on ERα+ breast cancer cells may also be linked to increased steroid hormone levels, which induce cellular proliferation and thus are an important factor for carcinogenesis (Folkerd and Dowsett, 2013). Indeed, RES treatment of mice (4 mg/kg i.p. for 7 days) resulted in an approximately twofold increase in E2 levels (El-Sayed and Bayan, 2015). Also a previous clinical study of post-menopausal women who were administered 1 g RES once a day for 12 weeks demonstrated a non-significant increase in serum E2 concentrations by 22.4% (Chow et al., 2014). Another study in healthy female volunteers (Chow et al., 2010) reported RES-associated menstrual changes as an adverse event in 4.8% of the subjects after oral consumption (1 g once a day for 4 weeks); again indicating altered steroid hormone levels.

We therefore hypothesized that RES may increase the level of active E2 in a dose-dependent manner, either by increasing the concentration of estrogen precursor steroids, or via inhibition of the biotransformation of E2 to conjugated metabolites, which do not promote ER-mediated activity (Samavat and Kurzer, 2015). Our hypothesis was supported by several previous *in vitro* and *in vivo* studies, which showed that RES inhibits various enzymes involved in the metabolism of estrogens, including 3β-hydroxysteroid dehydrogenase (3β-HSD), cytochrome P450 3A4 (CYP3A4), sulfotransferases (SULTs) and UDP-glucuronosyltransferases (UGTs) (Chan and Delucchi, 2000; Mesía-Vela and Kauffman, 2003; Furimsky et al., 2008; Chow et al., 2010; Mohamed and Frye, 2011; Li et al., 2014).

Therefore, the aim of the present study was to investigate the impact of RES on steroid metabolism in human ERα- MDA-MB-231 and ERα+ MCF-7 breast cancer cells. For this purpose, a specific and sensitive LC–HRMS assay was conducted to simultaneously quantify the 10 main steroids of the estrogenic metabolic pathway (Poschner et al., 2017). Differences in metabolism should be correlated with cell proliferation, which may explain the observed tumor-promoting effect of RES in ERα+ breast cancer.

## Materials and Methods

### Materials

4-Androstene-3,17-dione, 16α-hydroxy-17β-estradiol, 17β-estradiol, 17β-estradiol-3-*O*-(β-D-glucuronide) sodium salt, dehydroepiandrosterone, dehydroepiandrosterone-2,2,3,4,4,6-d6, dehydroepiandrosterone-3-*O*-sulfate, dehydroepiandrosterone-3-*O*-sulfate-2,2,3,4,4,6-d_6_-sodium salt, estrone and testosterone, as well as acetic acid, acetonitrile, ammonium acetate, dimethylsulfoxide, and *trans*-resveratrol, were obtained from Merck KGaA (Darmstadt, Germany). 17β-estradiol-3-*O*-sulfate sodium salt and estrone-3-*O*-sulfate sodium salt were purchased from Steraloids, Inc. (Newport, RI, United States). 4-Androstene-3,17-dione-2,2,4,6,6,16,16-d_7_ (AD-d_7_), 16α-hydroxy-17β-estradiol-2,4,17-d_3_ (E3-d_3_), 17β-estradiol-2,4,16,16-d_4_ (E2-d_4_), 17β-estradiol-16,16,17-d_3_-3-*O*-(β-D-glucuronide) sodium salt (E2-G-d_3_), 17β-estradiol-2,4,16,16-d_4_-3-*O*-sulfate sodium salt (E2-S-d_4_), estrone-2,4,16,16-d_4_ (E1-d_4_), estrone-2,4,16,16-d_4_-3-*O*-sulfate sodium salt (E1-S-d_4_), and testosterone-2,2,4,6,6-d_5_ (T-d_5_) were obtained from C/D/N Isotopes, Inc. (Pointe-Claire, QC, Canada). *Trans*-resveratrol-3-*O*-(β-D-glucuronide) sodium salt, *trans*-resveratrol-3-*O*-sulfate sodium salt, *trans*-resveratrol-4′-*O*-(β-D-glucuronide) sodium salt, *trans*-resveratrol-4′-*O*-sulfate sodium salt, and *trans*-resveratrol-3-*O*-4′-*O*-disulfate disodium salt were purchased from Santa Cruz Biotechnology, Inc. (Dallas, TX, United States). Purified water was obtained using an arium pro ultrapure water system (Sartorius AG, Göttingen, Germany).

### Cell Proliferation Studies

MDA-MB-231 and MCF-7 breast cancer cells were purchased from the American Type Culture Collection (ATCC; Manassas, VA, United States) and routinely cultivated at 37°C (95% humidity and 5% CO_2_) in phenolred-free Dulbecco’s modified Eagle medium F-12 (DMEM/F-12), fortified with 1% PenStrep^®^-solution and 10% fetal bovine serum (Invitrogen; Thermo Fisher Scientific, Inc., Waltham, MA, United States). All experiments were performed during the exponential growth phase of both cell lines. For the experiments, cells were seeded in 6-well-plates at a density of 1.0 × 10^6^ cells per well and allowed to attach for 24 h. Prior to incubation with RES or hormone precursors (DHEA and E1), the cells were washed twice with Dulbecco’s phosphate-buffered saline (DPBS; Invitrogen), and DMEM/F-12, containing 10% HyClone^®^ heat-inactivated charcoal-stripped fetal bovine serum (GE Healthcare Life Sciences, Logan, UT, United States), was subsequently added to exclude the interference of external hormones.

To elucidate the influence of RES, DHEA and E1 on MDA-MB-231 and MCF-7 cell proliferation, cells were incubated for 48 h with RES (0–100 μM), DHEA (0–100 nM), and E1 (0–100 nM), respectively. RES, DHEA, and E1 were dissolved in sterile-filtered DMSO prior to their addition to the cell medium to give a final DMSO concentration of 0.1%. Prior to cell counting with a Casy^®^ TT Cell Counter (OLS OMNI Life Science, Bremen, Germany), the supernatant medium was removed and cells were detached using 400 μl TrypLe^®^ solution (Invitrogen). All experiments were performed in triplicate, and the data were reported as the means ± standard deviation of all values.

### Metabolism of RES in MDA-MB-231 and MCF-7 Cells

MDA-MB-231 and MCF-7 cells were cultivated as described above and incubated with increasing concentrations of RES (0–100 μM). After 48 h, the cellular media (100 μl) were mixed with 200 μl ice-cold (-20°C) methanol and subsequently centrifuged (14000 rpm, 5 min). The clear supernatants were then diluted 1:1 with aqueous ammonium acetate buffer (5 mM, pH = 7.4), and 80 μl of the samples were injected onto the HPLC column. RES and its five glucuronidated and sulfated biotransformation products were quantified by HPLC, as described previously (Riha et al., 2014), using a Dionex UltiMate 3000 system (Sunnyvale, CA, United States) equipped with an L-7250 injector, an L-7100 pump, an L-7300 column oven (set at 15°C), a D-7000 interface and an L-7400 UV detector (Thermo Fisher Scientific, Inc.) set at a wavelength of 307 nm. Calibration of the chromatogram was accomplished using the external standard method. Linear calibration curves were produced by spiking drug-free DMEM/F-12 medium with standard solutions of RES, RES-3G, RES-3S, RES-4G, RES-4S, and RES-DS to give a concentration range from 0.001 to 10.0 μg/ml (mean correlation coefficients: >0.999). For this method, the lower limits of quantification (LLOQs) for RES, RES glucuronides and RES sulfates were 2.5, 10.1, and 4.0 ng/ml, respectively. The coefficients of accuracy and precision for these compounds were <11%.

### Inhibition of Steroid Metabolism by RES

Both cell lines were cultivated in the presence of HyClone^®^ heat-inactivated charcoal-stripped fetal bovine serum as described above and then treated with increasing concentrations of DHEA or E1 (0–100 nM), respectively, in the absence and presence of RES (0–100 μM). After 48 h (preliminary experiments showed the linearity of metabolite formation for this time-span), 2000 μl media aliquots were mixed with 20 μl deuterated internal standard solution and pre-cleaned using SPE on Oasis HLB 1 cc SPE cartridges (30 mg; Waters Corporation, Milford, MA, United States), as described previously (Poschner et al., 2017). Briefly, cartridges were preconditioned with 2 × 1.0 ml acetonitrile and 3 × 1.0 ml ammonium acetate buffer (10 mM, pH = 5.0), and the samples were loaded onto the columns. After washing with 1 × 1.0 ml ammonium acetate buffer (10 mM, pH = 5.0) and 2 × 1.0 ml acetonitrile/ammonium acetate buffer (10 mM, pH 5.0) 10:90 (v/v), the analytes were eluted using 2 × 650 μl acetonitrile/ammonium acetate buffer (10 mM, pH = 5.0) 95:5 (v/v) and evaporated until dry. Subsequently, samples were reconstituted in 270 μl acetonitrile/ammonium acetate buffer (10 mM, pH = 5.0) 25:75 (v/v) and stored at -80°C until further LC–HRMS analysis.

After media collection, cell monolayers were washed five times with 2.0 ml DPBS, detached using 400 μl TrypLE^®^ solution (37°C, 5 min), mixed with 600 μl DPBS and transferred into sample vials. Aliquots of these suspensions (100 μl each) were diluted and counted using the Casy^®^ TT Cell Counter to determine the exact number of cells per sample well. The remaining cell suspensions (900 μl each) were gently centrifuged (1000 rpm, 8 min), and the supernatants were discarded. The cell pellets were subsequently resuspended in 100 μl aqueous ammonium acetate buffer (10 mM, pH = 5.0) and lysed by five freeze-thaw-cycles in liquid nitrogen (3 min each), followed by thawing at ambient temperature. Ammonium acetate buffer (1000 μl) was added, and the suspensions were centrifuged (14000 rpm, 5 min). Subsequently, the supernatants were concentrated using the same SPE protocol as described above. All processed samples were then stored at -80°C until further LC–HRMS analysis. For each condition, three biologically independent experiments were performed; the reported values represent the overall means ± SD of all values.

### Steroid Hormone Quantification Using LC–HRMS

The 10 predominant metabolites of the estrogenic metabolic pathway (AD, DHEA, DHEA-S, E1, E1-S, E2, E2-S, E2-G, E3, and T) were then quantified using a selective and sensitive LC–HRMS assay, which was validated according to the ICH Q2(R1) guidelines, as described previously (Poschner et al., 2017). LC was performed with an UltiMate 3000 RSLC-series system coupled to a maXis HD ESI-Qq-TOF mass spectrometer (Bruker Corporation, Bremen, Germany). A Phenomenex Luna^®^ 3 μm C18(2) 100 Å LC column (250 mm × 4.6 mm I.D.; Phenomenex, Inc., Torrance, CA, United States), preceded by a Hypersil^®^ BDS-C18 guard column (5 μm, 10 mm × 4.6 mm I.D.; Thermo Fisher Scientific, Inc.) was used for the separation of the analytes at a flow rate of 1.0 ml/min and a temperature of 43°C. The injection volume was set to 100 μl for each sample; aqueous ammonium acetate buffer (10 mM, pH = 5.0) was used as solvent A, and acetonitrile as solvent B. The gradient was as follows: 25% solvent B at 0 min, 56.3% solvent B at 19 min, a washing step at 90% solvent B from 19.5 to 24.0 min, and column re-equilibration with 25% solvent B from 24.5 to 30.5 min. The ESI ion source settings were as follows: capillary voltage: -4.5 kV; dry gas flow rate: 8.0 l/min N_2_; nebulizer: 1.0 bar N_2_; and dry temperature: 200°C. The ion transfer parameters were set to 400 V_pp_ funnel RF and 300 V_pp_ multipole RF, the quadrupole ion energy was 8.0 eV, and the collision cell parameters were as follows: collision RF, 1100 V_pp_; collision energy, 10.0 eV; transfer time, 38 μs; and pre-pulse storage, 18 μs. In the range of *m/z* 150–500, full-scan mass spectra were recorded. Quality control samples, containing each analyte at a concentration of 6-, 60-, or 600-fold of the respective LLOQs, were analyzed in triplicate with each LC batch to ensure accurate quantification results (Supplementary Figure S1). The LLOQs for all 10 analytes, defined as the concentrations where the signal to noise ratio (S/N) is ≥9, were determined as follows: AD: 74.9 pg/ml; DHEA: 1904.0 pg/ml; DHEA-S: 8.0 pg/ml; E1: 19.0 pg/ml; E1-S: 4.0 pg/ml; E2: 140.9 pg/ml; E2-G: 12.0 pg/ml; E2-S: 3.4 pg/ml; E3: 28.4 pg/ml; and T: 54.1 pg/ml.

### Real-Time PCR Analysis

MCF-7 breast cancer cells were seeded in 6-well plates at a density of 1.0 × 10^6^ cells per well and allowed to attach overnight. Next day, cells were treated in kinetics (2, 4, 24, and 48 h, respectively) with 10 μM RES or with DMSO for the control samples (0 h time-point). Expression profiling of genes of interest was performed as detailed previously (Mechtcheriakova et al., 2007; Meshcheryakova et al., 2016). Upon treatment, total RNA was isolated using peqGOLD TriFAST^TM^ reagent (VWR, Vienna, Austria) according to the manufacturer’s protocol. 1 μg RNA was used for cDNA generation using the High Capacity cDNA Reverse Transcription Kit (Thermo Fisher Scientific, Inc.) according to the instructions of the manufacturer. Real-time PCR analysis was performed in the 96-well plate format on a QuantStudio 12K Flex Real-Time PCR System (Thermo Fisher Scientific, Inc.). *SULT1A1* and *SULT2A1* were detected using the corresponding TaqMan Assays (Thermo Fisher Scientific, Inc.). *UBC*, *GAPDH*, and *ACTB* were used as HKGs for normalization, based on the selection for the most stable expression during the treatment conditions among four pre-tested HKGs (*ACTB*, *UBC*, *GAPDH*, and *HPRT*). HKGs primers were self-designed using the Primer Express 3.0 software (Thermo Fisher Scientific, Inc.) and validated using the Human Total RNA Master Panel (TaKaRa Bio, Inc., Saint-Germain-en-Laye, France), as described before (Mechtcheriakova et al., 2007, 2011); Primers: *UBC* forward: ATTTGGGTCGCAGTTCTTG; *UBC* reverse: TGCCTTGACATTCTCGATGGT; *GAPDH* forward: CGGGTCAACGGATTTGGTC; *GAPDH* reverse: TGGCAACAATATCCACTTTACCAG; *ACTB* forward: AGGCACCAGGGCGTGAT; *ACTB* reverse: TGTAGAAGGTGTGGTGCCAGATT. For relative quantification, data were analyzed by applying the ΔΔCT method. Expression levels of target genes were normalized to the average of the HKGs and shown relative to unstimulated cells (0 h time-point). Gene(s) with Ct > 36 are classified herein as *not expressed*; gene(s) with Ct > 30 are classified as *low expressing gene(s)*. Using the GENEVESTIGATOR and the Affymetrix Human Genome U133 Plus 2.0 Array web based analysis platforms (Hruz et al., 2008), *SULT1E1* was found to be not expressed in MCF-7 cells and was therefore not assessed by real-time PCR.

### Data Analysis and Statistics

Compass DataAnalysis 4.2 and QuantAnalysis 2.2 software (Bruker Corporation) were used to analyze the acquired LC–HRMS data. For each analyte and internal standard pair, EICs were created, from which the respective peak areas were determined and the analyte/internal standard ratios were calculated for quantification.

Kinetic analyses of RES metabolism and steroid metabolism in the presence and absence of RES in both cell lines were performed using GraphPad Prism 6.0 software (GraphPad Software, Inc., La Jolla, CA, United States). Kinetic parameters for the formation of RES metabolites were best fitted to the substrate inhibition model: V = V_max_/(1 + K_m_/[S] + [S]/K_i_), or the sigmoidal Hill model: V = V_max_ × [S]^n^/([S_50_]^n^ + [S]^n^), whereas kinetics regarding steroid hormone metabolism were better estimated using the Michaelis–Menten model: V = V_max_ × [S]/(K_m_ + [S]), where V is the rate of the reaction, V_max_ is the maximum reaction velocity, K_m_ is the Michaelis constant, [S] is the initial substrate concentration, K_i_ is the inhibition constant, n is the Hill slope and S_50_ the concentration of substrate that produces a half-maximal enzyme velocity. The modes of inhibition were subsequently determined from Lineweaver–Burk plots, and the corresponding K_i_ values were calculated from Dixon plots using GraphPad Prism 6.0.

The same software package was also used for all statistical analyses. All values were expressed as the means ± SD of three independent biological replicates and one-way ANOVA combined with Tukey’s post-test were used to compare differences between control samples and treatment groups. The statistical significance threshold was defined as *P* < 0.05 for all calculations.

## Results

### Effects of RES on the Proliferation of MDA-MB-231 and MCF-7 Cells

**FIGURE 1:**
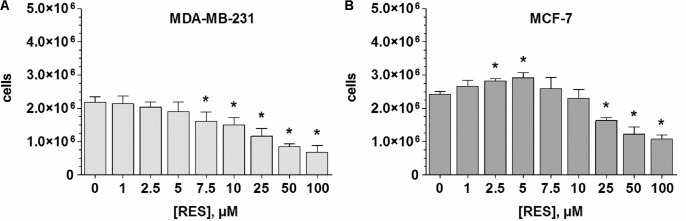
Influence of RES on MDA-MB-231 and MCF-7 cell proliferation. **(A)** MDA-MB-231 or **(B)** MCF-7 cells were incubated for 48 h with increasing concentrations of RES (0–100 μM) in steroid-deprived conditions. All data represent the means ± SD of three independent biological replicates. ^∗^*P* < 0.05 vs. untreated control samples.

To evaluate the effects of RES on breast cancer cell growth, ERα- MDA-MB-231 and ERα+ MCF-7 cells were exposed to increasing concentrations of RES (0–100 μM) in the absence of DHEA and E1. As shown in **Figure 1A**, RES inhibited the cell growth of MDA-MB-231 cells in a concentration-dependent manner. Even after the addition of 2.5 μM RES, the number of viable cells decreased non-significantly from 2.17 ± 0.18 × 10^6^ to 2.03 ± 0.16 × 10^6^ cells, but 100 μM RES resulted in a significant reduction by 68.6 ± 4.7%. Contrary to the MDA-MB-231 cells, RES demonstrated a biphasic effect in MCF-7 cells, as concentrations below 10 μM stimulated cellular growth with a maximal effect by 21.2 ± 3.3% to 2.92 ± 0.16 × 10^6^ cells at 5 μM RES (**Figure 1B**). A further increase of RES concentration (>10 μM), however, led to a significant inhibition of cell growth (1.08 ± 0.11 × 10^6^ cells at 100 μM RES). Interestingly, the inhibitory effect of RES was more evident in MDA-MB-231 compared with MCF-7 cells, as indicated by lower IC_50_ values (15.1 ± 4.9 vs. 37.4 ± 14.5 μM).

### RES Metabolism by MDA-MB-231 and MCF-7 Cells

In order to investigate whether the observed differences in the proliferation of MDA-MB-231 and MCF-7 cells by RES were due to alterations in RES metabolism, both cell lines were screened for the formation of the five major human conjugated RES metabolites. In addition to native RES, only RES-3G and RES-3S could be quantified in both cell lines, as the levels of RES-4G, RES-4S, or RES-DS formation were below the detection limit.

**FIGURE 2:**
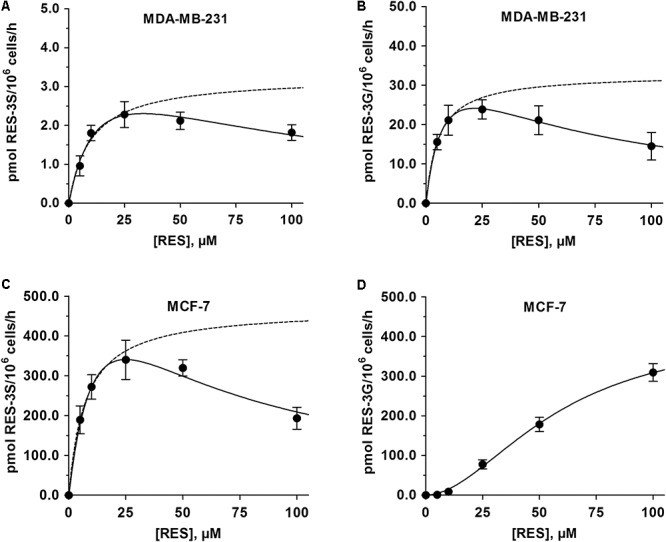
Kinetic profiles for the formation of 3-O-conjugated RES-metabolites by MDA-MB-231 and MCF-7 cells. The formation of **(A)** RES-3S and **(B)** RES-3G by MDA-MB-231 cells was determined following incubation with 0–100 μM RES for 48 h, and modeled using the substrate inhibition model. The formation of **(C)** RES-3S and **(D)** RES-3G by MCF-7 cells were evaluated with the same protocol. While the formation of RES-3S correlated with the substrate inhibition model, the formation of RES-3S was best described by the sigmoidal Hill model. Dashed lines represent the kinetic profiles without substrate inhibition. All data represent the means ± SD of three independent biological replicates.

In MDA-MB-231 cells, the maximum metabolite formation was observed following incubation with 25 μM RES, with a notable preference for glucuronidation (23.9 ± 2.4 pmol RES-3G/10^6^ cells/h) compared with sulfation (2.28 ± 0.33 pmol RES-3S/10^6^ cells/h) (**Figures 2A,B**). At higher RES concentrations, the formation of both RES conjugates decreased, best fitting to the substrate inhibition model, with K_i_ values of 54.0 ± 5.3 μM for RES-3G and 76.3 ± 35.2 μM for RES-3S.

Compared with MDA-MB-231 cells, the formation of RES-3S in MCF-7 cells was increased by 150-fold, with a mean maximum formation rate of 340.1 ± 49.4 pmol RES-3S/10^6^ cells/h (**Figure 2C**). As observed in MDA-MB-231 cells, treatment with RES up to 100 μM led to a pronounced reduction in RES-3S by 43.2%, again indicating substrate inhibition (K_i_: 39.4 ± 14.3 μM). Glucuronidation of RES in MCF-7 cells was also higher (10-fold increase), demonstrating a sigmoidal Hill kinetic pattern, with a maximum formation rate of 309.4 ± 22.2 pmol RES-3G/10^6^ cells/h at 100 μM RES (**Figure 2D**). Individual kinetic parameters for RES-S and RES-G in both cell lines are presented in **Table 1**.

**Table 1:** Kinetic parameters of RES metabolism by MDA-MB-231 and MCF-7 cells.

Cell line	Metabolite	Model	Parameters				
			K_m_ (μM)	V_max_ (pmol/10^6^ cells/h)	Slope (n)	R^2^	K_i_ (μM)
MDA-MB-231	RES-3S	Michaelis–Menten	9.83 ± 3.18	3.25 ± 0.44	n.a.	0.9395	n.a.
		Substrate inhibition	14.4 ± 5.6	4.31 ± 0.97	n.a.	0.9873	76.3 ± 35.2
	RES-3G	Michaelis–Menten	5.55 ± 3.43	32.8 ± 8.6	n.a.	0.9522	n.a.
		Substrate inhibition	8.61 ± 0.85	43.4 ± 2.2	n.a.	0.9992	54.0 ± 5.3
MCF-7	RES-3S	Michaelis–Menten	7.30 ± 0.44	468.4 ± 9.9	n.a.	0.9991	n.a.
		Substrate inhibition	8.19 ± 0.56	501.4 ± 17.1	n.a.	0.9889	39.4 ± 14.3
	RES-3G	Hill	58.5 ± 6.2	422.9 ± 38.9	1.86 ± 0.18	0.9988	n.a.

*Kinetic parameters were calculated using GraphPad Prism 6.0 software following the incubation of the cells with increasing concentrations of RES (0–100 μM) for 48 h. All data represent the means ± SD of three independent biological replicates. n.a., not applicable*.

### Effects of DHEA and E1 on the Proliferation of MDA-MB-231 and MCF-7 Cells

**FIGURE 3:**
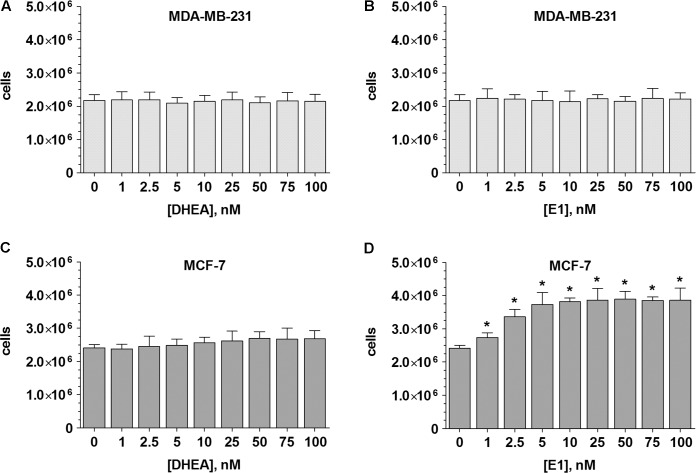
Influence of the hormone precursors DHEA and E1 on MDA-MB-231 and MCF-7 cell proliferation. MDA-MB-231 cells were incubated with increasing concentrations (0–100 nM) of **(A)** DHEA or **(B)** E1 for 48 h. MCF-7 cells were incubated with increasing concentrations (0–100 nM) of **(C)** DHEA or **(D)** E1 for 48 h. All data represent the means ± SD of three independent biological replicates.^∗^*P* < 0.05 vs. untreated control samples.

ERα- MDA-MB-231 and ERα+ MCF-7 breast cancer cells were cultured in a hormone-deprived environment for 48 h. In these conditions, the number of MDA-MB-231 cells doubled from 1.0 × 10^6^ to 2.17 ± 0.18 × 10^6^. A comparable increase in cell proliferation was also observed in the MCF-7 cell line (final cell count: 2.41 ± 0.94 × 10^6^ cells). Incubation of ERα- MDA-MB-231 cells with either DHEA or E1 (0–100 nM) did not further increase cell growth (**Figures 3A,B**). In ERα+ MCF-7 cells, the presence of DHEA only led to a minor, non-significant increase in cell numbers, to 2.69 ± 0.24 × 10^6^ cells at 100 nM (**Figure 3C**). Incubation with E1, however, significantly stimulated the proliferation of MCF-7 cells by 59.8%, to 3.85 ± 0.36 × 10^6^ cells at 100 nM (**Figure 3D**), confirming the hormone-dependency of this cell line.

### DHEA and E1 Metabolism by MDA-MB-231 and MCF-7 Cells

**FIGURE 4:**
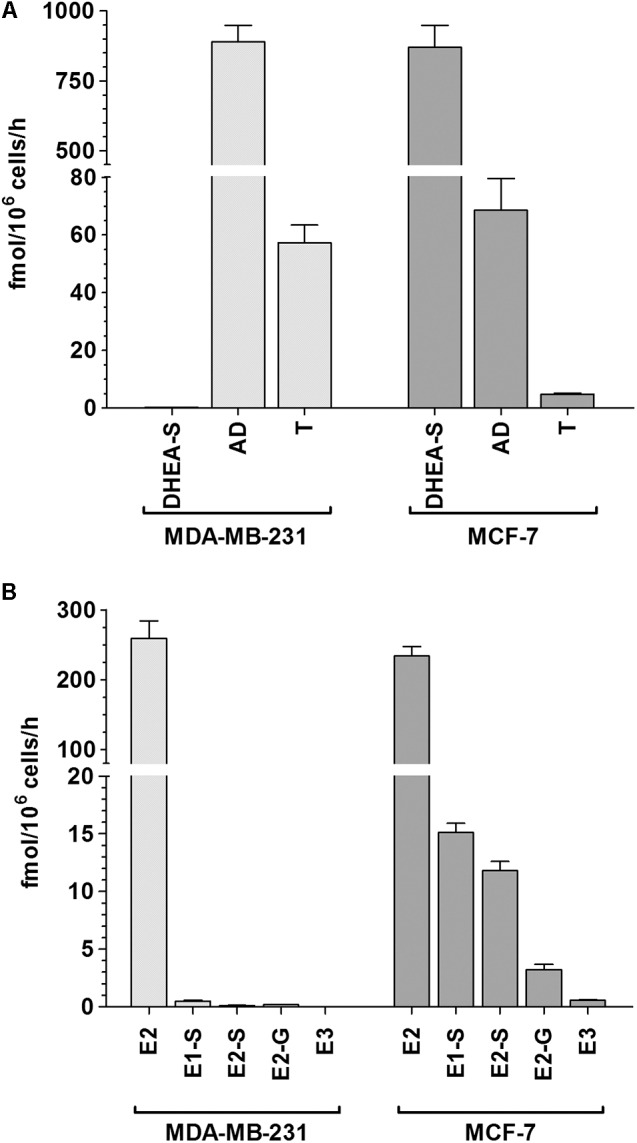
Patterns of steroid biotransformation rates in MDA-MB-231 and MCF-7 cells. MDA-MB-231 and MCF-7 cells were incubated with **(A)** 100 nM DHEA or **(B)** 100 nM E1 as hormone precursors, and media aliquots were analyzed for steroid metabolites after 48 h. All data represent the means ± SD of three independent biological replicates.

To assess differences in the metabolism of steroids by MDA-MB-231 and MCF-7 cells, both cell lines were incubated in the presence and absence of DHEA (100 nM) as a hormone precursor, and the formation of the nine major biotransformation products, namely DHEA-S, AD, T, E1, E1-S, E2, E2-S, E2-G and E3, was investigated by using a specific and sensitive LC–HRMS assay. The control samples (containing DMSO only) were performed for both cell lines to evaluate a possible endogenous steroid formation; however, neither in MCF-7 nor in MDA-MB-231 cells any endogenous steroid metabolites could be detected. Upon addition of 100 nM DHEA, the three metabolites DHEA-S, AD, and T could be quantified besides native DHEA in the supernatants of both cell lines; all other metabolites were below the LLOQ (**Figure 4A**).

In MDA-MB-231 cells, we observed that the 3β-HSD-mediated formation of AD was evidently favored (mean formation rate: 888.9 ± 60.1 fmol/10^6^ cells/h). AD was further metabolized via 17β-HSD to T, however, to a significantly lower extent (57.1 ± 6.2 fmol/10^6^ cells/h). The sulfation of DHEA was negligible, as the formation rate of DHEA-S was only 0.14 ± 0.02 fmol/10^6^ cells/h. Concomitant with the formation of these metabolites, DHEA concentration in the medium decreased by 26.8% from 100 to 73.2 ± 2.5 nM after 48 h. The total molar proportion of AD, T, and DHEA-S was 26.2%, indicating that these three biotransformation products represent almost 100% of all metabolites formed from the precursor DHEA in MDA-MB-231 cells (unmetabolized DHEA + total detected metabolites: 99.4%).

In MCF-7 cells, the formation rates of DHEA-S, AD, and T notably differed from MDA-MB-231 cells. DHEA-S was now the primary metabolite (mean formation rate: 870.0 ± 79.1 fmol/10^6^ cells/h), whereas the formation of AD and T was less pronounced (68.5 ± 11.1 and 4.78 ± 0.38 fmol/10^6^ cells/h, respectively). Concomitantly, the remaining DHEA concentration decreased by 54.9% from 100.0 to 45.1 ± 1.8 nM, which once again reflected the total molar proportion of these three metabolites (54.7%).

To further evaluate the formation rates of estrogens and their respective conjugates, MDA-MB-231 and MCF-7 cells were also incubated in the presence and absence of 100 nM E1 as a hormone precursor (**Figure 4B**). Again, control samples (containing DMSO only) revealed no endogenous formation of estrogen metabolites in both cell lines. E2 was identified as the predominant metabolite with comparable formation rates in MDA-MB-231 and MCF-7 cells (258.9 ± 25.1 fmol/10^6^ cells/h vs. 233.9 ± 13.4 fmol/10^6^ cells/h). The hydroxylation of E2 to E3 was a minor pathway in MCF-7 cells (0.57 ± 0.06 fmol/10^6^ cells/h), and was below the LLOQ in MDA-MB-231 cells. However, the formation rates of steroid conjugates differed markedly between both cell lines. While the formation of E1-S, E2-S and E2-G were negligible in MDA-MB-231 cells (E1-S: 0.49 ± 0.07 > E2-G: 0.18 ± 0.02 > E2-S: 0.12 ± 0.02 fmol/10^6^ cells/h), the total conjugation of E1 and E2 was up to 40-fold higher in MCF-7 cells (E1-S: 15.1 ± 0.8 > E2-S: 11.8 ± 0.8 > E2-G: 3.23 ± 0.45 fmol/10^6^ cells/h). In both cell lines, the total molar proportions of all detected metabolites amounted to 12.0% and 12.8%, with a concomitant decrease of native E1 by 12.3% and 12.9%, respectively.

The kinetic profiles for the formation of DHEA and E1 metabolites by MDA-MB-231 and MCF-7 cells were then evaluated over a DHEA and E1 concentration range of 0–100 nM for 48 h. The formation kinetics of DHEA-S, AD, T, E1-S, E2, E2-S, and E2-G best fitted to a hyperbolic Michaelis–Menten model, with the highest V_max_ values for AD in MDA-MB-231 cells (1994.0 ± 364.5 fmol/10^6^ cells/h) and for DHEA-S in MCF-7-cells (2873.0 ± 327.4 fmol/10^6^ cells/h). K_m_ values in both cell lines were within a similar range for AD, E2, E1-S and E2-G, but were significantly lower for DHEA-S, T and E2-S in MDA-MB-231 cells, compared with in MCF-7 cells (**Table 2**). The kinetic parameters for the formation of E3 could not be calculated, as only the highest concentration of E1 (100 nM) resulted in concentrations of E3 above the LLOQ for this assay.

**Table 2:** Kinetic parameters of steroid metabolism by MDA-MB-231 and MCF-7 cells.

Hormone precursor	Metabolite	MDA-MB-231		MCF-7	
		K_m_ (nM)	V_max_ (fmol/10^6^ cells/h)	K_m_ (nM)	V_max_ (fmol/10^6^ cells/h)
DHEA	DHEA-S	55.4 ± 20.5	0.22 ± 0.04	**229.2 ± 52.2^∗^**	**2873.0 ± 327.4^∗^**
	AD	126.1 ± 36.6	1994.0 ± 364.5	114.6 ± 3.6	**147.4 ± 8.1^∗^**
	T	56.3 ± 21.4	91.2 ± 15.9	**107.5 ± 6.3^∗^**	**9.96 ± 1.42^∗^**
E1	E2	174.0 ± 94.1	703.0 ± 164.5	168.6 ± 8.7	628.6 ± 62.0
	E1-S	119.5 ± 68.1	1.08 ± 0.38	101.9 ± 22.9	**30.4 ± 2.5^∗^**
	E2-S	38.0 ± 13.6	0.16 ± 0.02	**113.8 ± 35.5^∗^**	**24.7 ± 4.9^∗^**
	E2-G	87.9 ± 32.2	0.33 ± 0.07	123.5 ± 8.4	**7.29 ± 1.36^∗^**

*Kinetic parameters were calculated using GraphPad Prism 6.0 software following the incubation of the cells for 48 h with increasing concentrations of DHEA or E1 (25–100 nM). All data represent the means ± SD of three independent biological replicates. Values in bold and marked with an asterisk (^∗^) are significantly different compared with MDA-MB-231 cells (*P* < 0.05)*.

### Inhibition of Conjugated DHEA and E1 Metabolites by RES

**FIGURE 5:**
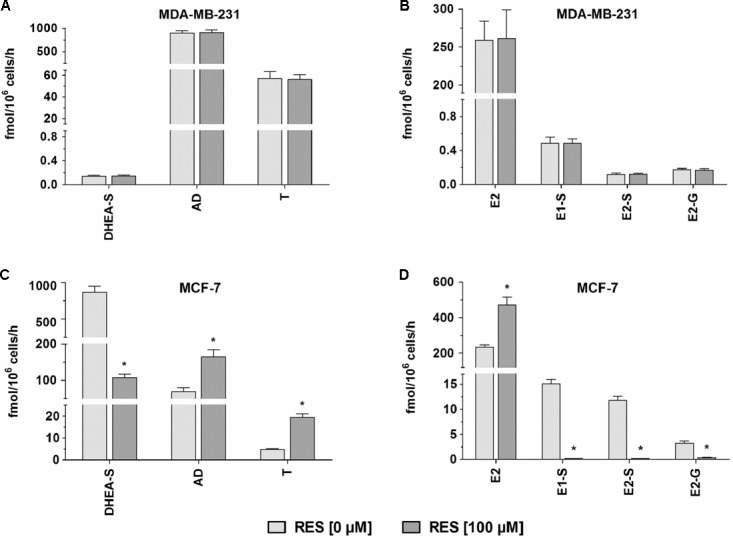
Effect of RES on steroid metabolism by MDA-MB-231 and MCF-7 cells. MDA-MB-231 cells were incubated with **(A)** 100 nM DHEA or **(B)** 100 nM E1 as a hormone precursor in the presence and absence of 100 μM RES for 48 h. MCF-7 cells were incubated with **(C)** 100 nM DHEA or **(D)** 100 nM E1 as a hormone precursor in the presence and absence of 100 μM RES for 48 h. All data represent the means ± SD of three independent biological replicates.^∗^*P* < 0.05.

To determine whether there was a possible inhibitory effect of RES on steroid metabolism, MDA-MB-231 and MCF-7 cells were treated with 100 nM DHEA or E1 for 48 h, in the presence or absence of 100 μM RES. As shown in **Figures 5A,B**, RES did not affect the formation of DHEA-S, E1-S, E2-S, and E2-G in MDA-MB-231 cells. Conversely, a marked effect of RES on the conjugation of DHEA, E1, and E2 was observed in MCF-7 cells (**Figures 5C,D**). The mean formation of DHEA-S, E1-S, E2-S, and E2-G was almost quantitatively reduced by 87.7%, 99.1%, 98.8%, and 89.7%, respectively.

**FIGURE 6:**
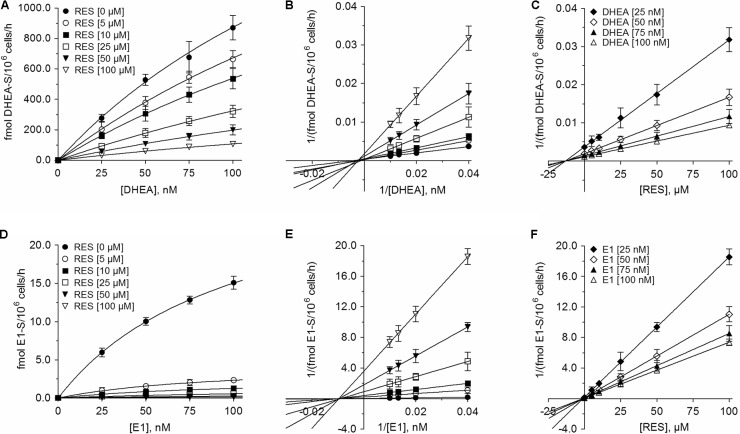
Kinetic profiles of the inhibition of DHEA sulfation and E1 sulfation in MCF-7 cells by RES. The kinetics of **(A–C)** DHEA sulfation and **(D–F)** E1 sulfation were calculated following the incubation of MCF-7 cells with 0–100 nM DHEA or E1 as hormone precursors in the absence or presence of 0–100 μM RES for 48 h. Data are displayed as Michaelis–Menten, Lineweaver–Burk, and Dixon plots. All data represent the means ± SD of three independent biological replicates.

**FIGURE 7:**
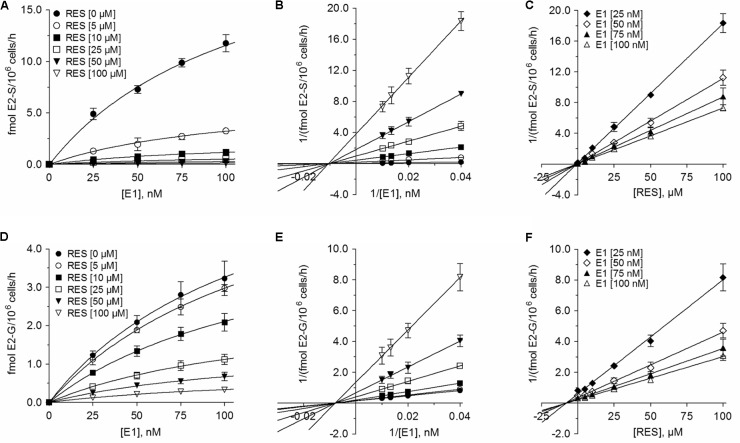
Kinetic profiles of the inhibition of E2 conjugations in MCF-7 cells by RES. The kinetics of **(A–C)** E2 sulfation and **(D–F)** E2 glucuronidation were calculated following the incubation of MCF-7 cells with 0–100 nM E1 as a hormone precursor in the absence or presence of 0–100 μM RES for 48 h. Data are displayed as Michaelis–Menten, Lineweaver–Burk, and Dixon plots. All data represent the means ± SD of three independent biological replicates.

The inhibition of all four metabolites best fitted to the Michaelis–Menten kinetic model (**Figures 6**, **7**). The V_max_ values for the formation of the conjugates were significantly decreased with increasing concentrations of RES, while the corresponding K_m_ values were virtually unaffected (**Table 3**). This indicated non-competitive inhibition by RES for all conjugates, which was further confirmed by Lineweaver–Burk and Dixon plots. The more pronounced inhibition of E1 and E2 sulfation compared with DHEA sulfation and E2 glucuronidation by RES was also associated with significantly lower K_i_ values (E2-S: 0.73 ± 0.07 μM < E1-S: 0.94 ± 0.03 μM < E2-G: 7.92 ± 0.24 μM < DHEA-S: 13.2 ± 0.2 μM), indicating decreased rates of E1-S and E2-S formation even at low RES concentrations.

**Table 3:** Kinetic parameters of steroid metabolism by MCF-7 cells in the absence and presence of RES.

Metabolite	K_m_ (nM)						V_max_ (fmol/10^6^ cells/h)					
	0 μM RES	5 μMRES	10 μMRES	25 μMRES	50 μMRES	100 μMRES	0 μMRES	5 μMRES	10 μMRES	25 μMRES	50 μMRES	100 μMRES
DHEA-S	329.2 ± 52.2	311.7 ± 71.9	323.7 ± 53.7	404.1 ± 93.1	402.8 ± 34.7	385.0 ± 46.6	2873.0 ± 57.4	2766.2 ± 53.9	**2280.1 ± 81.4^∗^**	**1797.6 ± 48.9^∗^**	**993.5 ± 74.8^∗^**	**522.5 ± 54.8^∗^**
E1-S	101.9 ± 12.9	94.0 ± 12.0	100.5 ± 8.6	120.5 ± 15.5	108.9 ± 4.1	105.4 ± 9.0	30.4 ± 0.5	**4.51 ± 0.33^∗^**	**2.54 ± 0.21^∗^**	**1.23 ± 0.11^∗^**	**0.57 ± 0.02^∗^**	**0.28 ± 0.01^∗^**
E2-S	103.8 ± 15.5	105.6 ± 15.9	99.3 ± 12.8	94.9 ± 14.7	100.6 ± 11.2	99.7 ± 6.0	24.7 ± 4.9	**6.64 ± 0.58^∗^**	**2.34 ± 0.19^∗^**	**1.01 ± 0.10^∗^**	**0.56 ± 0.01^∗^**	**0.27 ± 0.01^∗^**
E2-G	123.5 ± 8.4	135.0 ± 5.9	136.0 ± 6.0	131.5 ± 8.0	126.2 ± 13.2	131.6 ± 3.9	7.29 ± 0.36	7.00 ± 0.20	**4.98 ± 0.14^∗^**	**2.60 ± 0.12^∗^**	**1.51 ± 0.11^∗^**	**0.77 ± 0.02^∗^**
AD	114.6 ± 9.6	103.5 ± 10.4	101.9 ± 7.9	111.8 ± 10.7	106.3 ± 4.2	99.6 ± 8.2	147.4 ± 13.1	148.5 ± 9.4	**172.6 ± 7.7^∗^**	**242.0 ± 14.4^∗^**	**295.3 ± 9.4^∗^**	**328.8 ± 12.9^∗^**
T	107.5 ± 6.3	107.1 ± 5.9	107.5 ± 3.8	106.4 ± 2.4	106.0 ± 4.0	103.3 ± 4.8	9.95 ± 0.42	**13.5 ± 0.5^∗^**	**18.4 ± 0.7^∗^**	**26.7 ± 0.4^∗^**	**35.9 ± 0.8^∗^**	**39.7 ± 1.1^∗^**
E2	168.6 ± 11.7	146.0 ± 15.6	155.4 ± 9.7	152.9 ± 13.6	171.8 ± 14.3	174.3 ± 12.0	628.6 ± 72.0	**816.4 ± 76.6^∗^**	**1001.4 ± 43.0^∗^**	**1079.8 ± 71.4^∗^**	**1247.3 ± 56.2^∗^**	**1302.2 ± 64.6^∗^**

*Kinetic parameters were calculated using GraphPad Prism 6.0 software following the incubation of the cells with increasing concentrations of RES (0–100 μM) and DHEA or E1 (25 to 100 nM) as hormone precursors for 48 h. All data represent the means ± SD of three independent biological replicates. Values in bold and marked with an asterisk (^∗^) are significantly different in comparison to the control values (*P* < 0.05)*.

### Effect of RES on Unconjugated DHEA and E1 Metabolites

**FIGURE 8:**
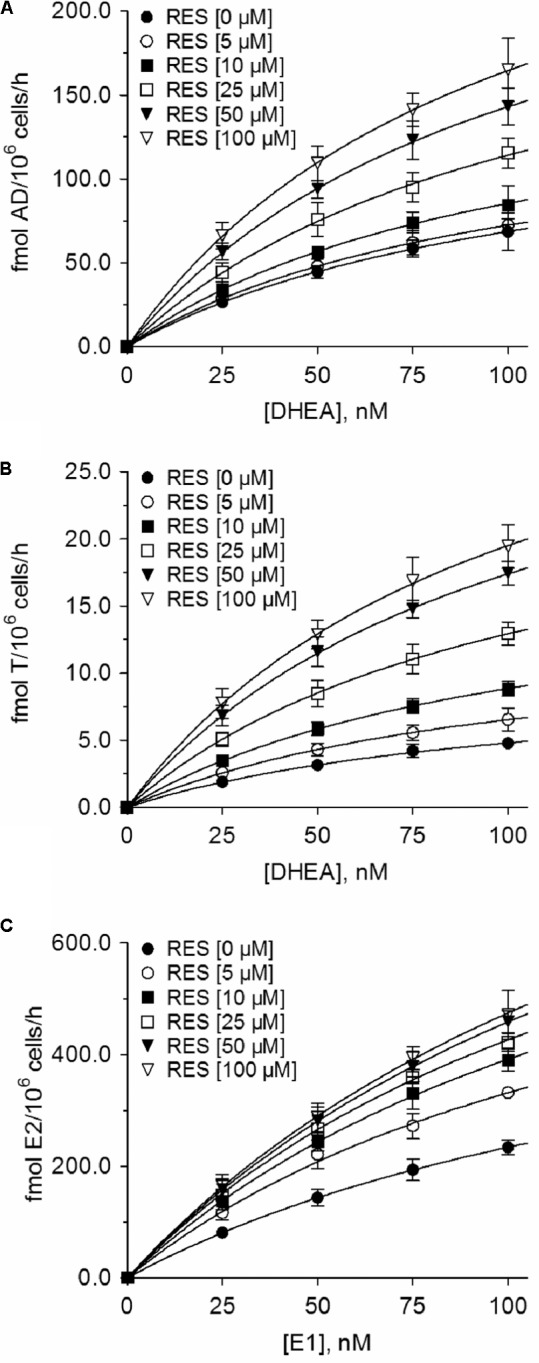
Kinetic profiles of the increase of unconjugated steroids in MCF-7 cells in the presence of RES. Michaelis–Menten plots for the formation of **(A)** AD, **(B)** T, and **(C)** E2 were calculated following the incubation of MCF-7 cells with 0–100 nM DHEA or E1 as hormone precursors in the absence or presence of 0–100 μM RES for 48 h. All data represent the means ± SD of three independent biological replicates.

In addition to the quantification of steroid conjugates, we also assessed the effects of RES treatment on the formation of unconjugated AD, T, and E2. Again, RES exerted no significant influence in MDA-MB-231 cells (**Figures 5A,B**); however, in MCF-7 cells, RES markedly increased the levels of these three metabolites (**Figures 5C,D**). 100 μM RES treatment led to a significant increase in AD concentrations by 141.0% (from 68.5 ± 11.1 to 165.1 ± 18.7 fmol/10^6^ cells/h), whereas T levels increased by 305.9% (from 4.78 ± 0.38 to 19.4 ± 1.6 fmol/10^6^ cells/h); unconjugated E2 concentrations also increased by 101.6% (from 233.9 ± 13.4 to 471.5 ± 43.6 fmol/10^6^ cells/h). The kinetic profiles for AD, T, and E2 in MCF-7 cells were then evaluated over a concentration range of 0–100 nM DHEA and E1, respectively. As observed for the inhibition of DHEA, E1, and E2 conjugates, the K_m_ values were not affected by RES treatment, whereas the V_max_ values increased in a concentration-dependent manner (**Figure 8** and **Table 3**), thereby confirming a stimulatory effect of RES on the formation of AD, T, and E2.

### Down-Regulation of SULT1A1 and SULT2A1 mRNA Expression in MCF-7 Cells by RES

Next, we raised the question whether RES regulates *SULT1A1* and *SULT2A1* on the transcriptional level during the time of treatment. Therefore, MCF-7 cells were treated with 10 μM RES for 2, 4, 24, and 48 h. We found that RES did not affect the transcription of *SULT1A1* for up to 4 h of treatment (Supplementary Figure S2). However, a moderate, but non-significant down-regulation on the mRNA level was observed after 24 and 48 h (23.6 ± 13.0 and 30.4 ± 11.0%, respectively). Expression levels of *SULT2A1* were low at time 0 h (Ct > 30) and again marginally down-regulated after 24 and 48 h (data not shown).

### Effect of RES in the Presence of E1 on the Proliferation of MDA-MB-231 and MCF-7 Cells

**FIGURE 9:**
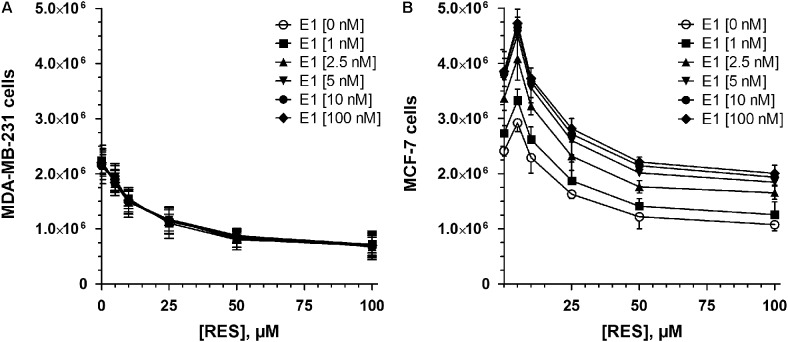
Effect of co-incubation with RES and E1 on cellular proliferation. **(A)** MDA-MB-231 and **(B)** MCF-7 breast cancer cells were incubated in the absence or presence of 0–100 μM RES with increasing concentrations of E1 (0–100 nM) as a hormone precursor. All data represent the means ± SD of three independent biological replicates.

The simultaneous incubation of MDA-MB-231 cells with RES and E1 up to 100 μM had no observable effect on cell proliferation (**Figure 9A**); however, in MCF-7 cells we observed a pronounced induction of cellular proliferation after co-incubation for 48 h (**Figure 9B**). Interestingly, the induction of cell growth was most pronounced at 5 μM RES, resulting in a twofold increase in cell counts after co-incubation with 100 nM E1, compared with the control samples in the absence of E1. The stimulatory effects of 100 nM E1 were also observed at higher RES concentrations up to 100 μM, thereby almost counteracting the observed anti-proliferative effects of RES in a hormone-deprived setting.

## Discussion

The role of RES in preventing breast cancer is controversial, as some studies have proposed it may increase its risk. Our data revealed that higher RES concentrations (up to 100 μM) significantly inhibited proliferation in both ERα- and ERα+ breast cancer cell lines, with IC_50_ values of 15.1 ± 4.9 μM for MDA-MB-231 cells and 37.4 ± 14.5 μM for MCF-7 cells. These data are in accordance with *in vivo* data from animal experiments (Garvin et al., 2006), which also reported a reduction of cancer xenograft growth in mice treated with 25 mg/kg RES per day for 3 weeks. The approximately 2.5-fold higher IC_50_ value of RES against MCF-7 cells may be partially explained by altered RES metabolism, as the formation of RES-3G and RES-3S was increased by up to 150-fold compared with MDA-MB-231 cells. Our data are supported by the findings of Murias et al. (2008), who also reported a distinct correlation between RES metabolism efficacy and cytotoxicity in various breast cancer cell lines.

However, at concentrations below 10 μM RES, the growth of ERα+ MCF-7 cells, but not ERα- MDA-MB-231 cells, was increased, with a maximal stimulatory effect of 21.2% at 5 μM RES compared with untreated controls. A similar effect was also reported by a recent *in vivo* study (Andreani et al., 2017), which reported an induction of ERα+ breast cancer growth at low RES concentrations in Δ16HER2 mice receiving 4 μg RES daily for 15 weeks.

These conflicting results with regards to RES may be attributed to altered estrogen levels in breast tissue, as breast cancer growth and progression are closely associated with female steroid hormones. As metabolism is the key factor for the alteration of cellular estrogens, we investigated, for the first time, the effects of RES on the metabolism of steroids within ERα- MDA-MB-231, and ERα+ MCF-7 human breast cancer cells. In the absence of RES, DHEA, and E1 induced no effect on the proliferation of ERα- MDA-MB-231 cells after 48 h. Conversely, in ERα+ MCF-7 cells, DHEA produced a slight increase and E1 a significant increase in cell proliferation, which once again indicated the hormone-dependency of this breast cancer cell line. Additionally, we screened for the formation of DHEA and E1 metabolites in both cell lines. After incubation with DHEA, three metabolites, namely DHEA-S, AD and T, were quantified in the supernatant media of both cell lines. Interestingly, the sulfation of DHEA was identified as the dominant pathway in MCF-7 cells (V_max_: 2873.0 ± 527.4 fmol/10^6^ cells/h), while in MDA-MB-231 cells, the formation levels of AD and T were approximately 10-fold higher compared with in MCF-7 cells; the formation of DHEA-S only amounted to a V_max_ value of 0.22 ± 0.04 fmol/10^6^ cells/h.

Subsequently, we also incubated both cell lines with E1 as a hormone precursor. LC–HRMS analyses revealed that E2 was the predominant metabolite, with V_max_ values of 703.0 ± 164.5 fmol/10^6^ cells/h and 628.6 ± 62.0 fmol/10^6^ cells/h for MDA-MB-231 and MCF-7 cells, respectively. While the formation of all other metabolites was almost negligible in MDA-MB-231 cells (V_max_ values < 1.1 fmol/10^6^ cells/h), E1-S, E2-S, E2-G, and E3 formation levels amounted to 11.6% of all biotransformation products in MCF-7 cells. CYP3A4-mediated hydroxylation of E2 to E3, however, was suggested to involve a minor metabolic pathway undertaken by MCF-7 cells, as the formation of E3 could not be quantified at E1 concentrations of < 100 nM. Sulfation was evidently the preferred pathway for E1 and E2 metabolism in MCF-7 cells, as the V_max_ values were significantly higher (E1-S: 30.4 ± 2.5 fmol/10^6^ cells/h and E2-S: 24.7 ± 4.9 fmol/10^6^ cells/h) compared with those for E2 glucuronidation (7.29 ± 1.36 fmol/10^6^ cells/h). These data were supported by previous *in vitro* investigations, which also revealed, based on significantly higher SULT expression, that the formation levels of E1-S and E2-S in ERα+ MCF-7 cells were more than sevenfold higher than in ERα- MDA-MB-231 cells after incubation with E1 for 24 h (Pasqualini, 2009). This may also occur in breast cancer patients, as higher SULT expression levels were also reported in ERα+ breast tumors compared with in ERα- breast cancer tissues (Adams et al., 1979).

**FIGURE 10:**
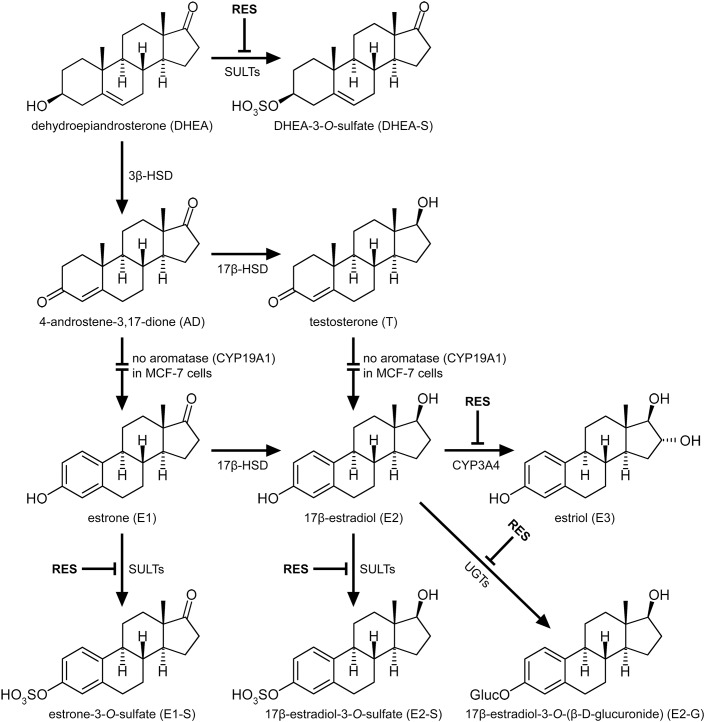
Interaction of RES with the metabolism of steroid hormones in MCF-7 breast cancer cells. RES inhibited the formation of the conjugated metabolites DHEA-S, E1-S, E2-S and E2-G, as well as the formation of E3, in a dose-dependent manner, thereby increasing the levels of AD, T, and active E2.

In the ERα- MDA-MB-231 cells, RES did not significantly affect the formation of DHEA and E1 metabolites; however, in the ERα+ MCF-7 cells, RES significantly inhibited the formation of DHEA-S, consequently increasing the pool of unconjugated DHEA and leading to increased AD and T levels. Contrary to breast tumor tissue, MCF-7 cells express low levels of aromatase (Zhou et al., 1993), explaining why AD and T were not metabolized to E1 and E2, respectively. The presence of aromatase in females may thus further increase both levels (**Figure 10**).

We therefore incubated MCF-7 cells with E1 as a hormone precursor in the presence of RES. As shown in **Figure 5**, RES significantly inhibited the formation of E1-S, E2-S and E2-G, thereby resulting in an approximate twofold increase in active E2 levels (**Figure 10**). Our data are in line with previous animal studies, which also observed that RES increased free E2 levels via the inhibition of E2 hydroxylation, glucuronidation, or sulfation in mice (Oskarsson et al., 2014) and rats (Banu et al., 2016). A non-significant increase in E2 serum concentrations (22.4%) was also reported in post-menopausal women receiving 1 g RES once a day for 12 weeks; however, as dansylchloride-derivatization was used for steroid quantification, the authors could not distinguish between free E2 and its conjugates (Chow et al., 2014).

This increase in unconjugated E2 may cause the observed induction of cell proliferation in ERα+ MCF-7 cells, particularly at RES concentrations < 10 μM. Similar findings have been reported by Chen et al. (2015), who also revealed that at physiological concentrations of E2, low doses of RES may stimulate the proliferation of ERα+ breast cancer cells. Therefore, increased E2 levels may be observed in hormone-dependent breast cancer patients following the intake of high-dose dietary supplements containing RES, as K_i_ values for the formation of E1-S and E2-S in MCF-7 breast cancer cells were 0.94 ± 0.03 and 0.73 ± 0.07 μM, respectively. The pronounced inhibition of estrogen sulfation by RES may also occur in other organs, as demonstrated by Furimsky et al. (2008) in S9 fractions from human liver (K_i_: 1.1 μM) and jejunum (K_i_: 0.6 μM).

In breast tumor tissue, steroid sulfation is catalyzed by SULT1E1, SULT1A1, and SULT2A1 (Mueller et al., 2015). Contrary to tumor tissue, MCF-7 cells do not express SULT1E1; steroid sulfation by this enzyme can be therefore excluded (Falany et al., 2002; Hruz et al., 2008). As the sulfation of E1, E2, and DHEA was strongly inhibited by RES in MCF-7 cells even upon addition of low concentrations, we also investigated a possible regulation of *SULT1A1* and *SULT2A1* mRNA expression after incubating MCF-7 cells with 10 μM RES. RES at this concentration showed an almost complete inhibition of E1 and E2 sulfation (91.6 ± 1.1% and 90.0 ± 1.0%, respectively, see **Figures 6D**, **7A**), but had no effect on the cellular proliferation (**Figure 1B**) even after 48 h. Possible candidates for RES mediating a down-regulation of estrogen sulfating enzymes are the AhR and the pregnane X receptor (PXR) (Casper et al., 1999; Kodama and Negishi, 2013; Moscovitz et al., 2018). Both receptors are known to regulate mRNA expression of estrogen-metabolizing enzymes including SULTs. Because RES is known to work as an antagonist for AhR and PXR and is capable of down-regulating both nuclear receptors and SULTs, RES might inhibit estrogen sulfation via these pathways.

We found only an insignificant down-regulation of *SULT1A1* mRNA by 30.4 ± 11.0% after longer-term application of 10 μM RES, suggesting that transcriptional regulation by these receptors might not contribute significantly to the observed reduction in the formation of steroid sulfates in the MCF-7 cells. These data might also implicate that breast cancer cell lines are different from the benign MCF-10A breast epithelial cells, where down-regulation of the AhR by RES significantly affect estrogen-metabolizing enzymes and cause a down-regulation of *SULT1E1*, particularly as *SULT1E1* is not expressed in MCF-7 cells (Licznerska et al., 2017). Further *in vitro* studies are therefore needed to elucidate the differences in the down-regulation of steroid metabolizing enzymes between benign and malign human breast cell lines.

Our data also showed that autophagy might not be induced in MCF-7 cells by 10 μM RES since cell proliferation was not affected. However, at higher RES concentrations, formation of autophagosomes might become important and was indeed observed by Scarlatti et al. (2008) in MCF-7 cells incubated with 64 μM RES for 48 h.

Based on our data, RES inhibits steroid sulfation in MCF-7 cells mainly via direct binding to SULT enzymes, thereby reducing their activities. This is supported by the Lineweaver–Burk plots in our study that clearly show non-competitive inhibition of E1, E2, and DHEA sulfation by 5–100 μM RES (**Figures 6B,E**, **7B**); both RES and the steroid substrates bind to the SULT enzymes at any given time forming an enzyme-substrate-inhibitor complex. Our observation is confirmed by the *in vitro* study of Ung and Nagar (2009), demonstrating RES-induced SULT1A1 and SULT1E1 inhibition in stably transfected MCF-7 cells.

Whether RES may accumulate to levels in human breast tissue necessary to inhibit DHEA, E1, and E2 metabolism remains unknown. Our recent study demonstrated that following oral application to rats (10 mg/kg), RES distributes to a variety of organs, including the liver, spleen, kidney, heart, lung and brain, strongly indicating the potential uptake of RES also into breast tissue (Böhmdorfer et al., 2017). Due to its low oral bioavailability, blood and tissue concentrations of RES following the dietary intake of red wine, peanuts or berries to inhibit estrogen metabolism may be markedly lower than our calculated K_i_ values, suggesting no significant effect on E2 levels. However, the daily high-dose administration of RES (0.5–5.0 g/day) for up to 29 days to 40 healthy subjects resulted in peak plasma concentrations of up to 4.24 μM (Brown et al., 2010). Additionally, the levels of RES-3S and RES-3G were approximately 10- and 6-fold higher, respectively. Whether RES glucuronides and sulfates also exhibit inhibitory effects toward DHEA, E1, and E2 conjugation remains to be investigated. Any inhibitory effects may further increase the plasma concentration of free E2 following high-dose RES supplementation, thereby inducing the progression of ERα+ breast cancer.

## Conclusion

The present study demonstrated the non-competitive inhibition of the steroid metabolomics pathway in ERα+ MCF-7 but not in ERα- MDA-MB-231 breast cancer cells by low micromolar concentrations of RES, which led to a significant, twofold increase of free E2, capable of stimulating the proliferation of ERα+ breast cancer cells. As the content of RES in food is relatively low, an increased risk of breast cancer progression may only be observed after the continuous consumption of high-dose RES supplements. Further long-term human studies simultaneously monitoring free estrogens and their conjugates are therefore highly warranted to evaluate the efficacy and safety of RES supplementation, particularly in patients diagnosed with ERα+ breast cancer.

## Author Contributions

SP performed all cell culture experiments, the LC–HRMS analysis and the data analysis, and contributed to the manuscript. AM-S analyzed the data and contributed to the manuscript. MZ, JW, and DD performed the LC–HRMS analyses. AM and DM performed the real-time PCR analyses and contributed to the manuscript together with TT. BP cultivated the breast cancer cell lines and performed the inhibition experiments. WJ supervised the experiments, analyzed the data, and wrote the final version of the manuscript.

## Conflict of Interest Statement

The authors declare that the research was conducted in the absence of any commercial or financial relationships that could be construed as a potential conflict of interest.

## Abbreviations

3β-HSD: 3β-hydroxysteroiddehydrogenase
17β-HSD: 17β-hydroxysteroiddehydrogenase
AD: 4-androstene-3,17-dione
AhR: aryl hydrocarbon receptor
cDNA: complementary deoxyribonucleic acid
CYP: cytochrome P450
DHEA: dehydroepiandrosterone
DHEA-S: dehydroepiandrosterone-3-*O*-sulfate
DMEM: Dulbecco’s modified Eagle’s medium
DMSO: dimethylsulfoxide
DPBS: Dulbecco’s phosphate-buffered saline
E1: estrone
E1-S: estrone-3-*O*-sulfate
E2: 17β-estradiol
E2-G: 17β-estradiol-3-*O*-(β-D-glucuronide)
E2-S: 17β-estradiol-3-*O*-sulfate
E3: estriol (16α-OH-17β-estradiol)
EIC: extracted ion chromatogram
ERα: estrogen-receptor alpha
ESI: electro-spray ionization
HKGs: housekeeping genes
K_i_: inhibition constant
K_m_: Michaelis constant
LC–HRMS: liquid chromatography–high resolution mass spectrometry
LLOQ: lower limit of quantification
mRNA: messenger ribonucleic acid
PCR: polymerase chain reaction
PXR: pregnane X receptor
RES: *trans*-resveratrol
RES-3G: *trans*-resveratrol-3-*O*-(β-D-glucuronide)
RES-3S: *trans*-resveratrol-3-*O*-sulfate
RES-4G: *trans*-resveratrol-4′-*O*-(β-D-glucuronide)
RES-4S: *trans*-resveratrol-4′-*O*-sulfate
RES-DS: *trans*-resveratrol-3-*O*-4′-*O*-disulfate
SD: standard deviation
SPE: solid phase extraction
SULT: sulfotransferase
T: testosterone
UGT: UDP-glucuronosyl transferase
V_max_: maximum reaction velocity.
