# Supplementary figures and images for: Resveratrol Inhibits Key Steps of Steroid Metabolism in a Human Estrogen-Receptor Positive Breast Cancer Model: Impact on Cellular Proliferation
Source: Front Pharmacol. 2018 Jul 10;9:742. doi: 10.3389/fphar.2018.00742 (PMC6048268; doi:10.3389/fphar.2018.00742)

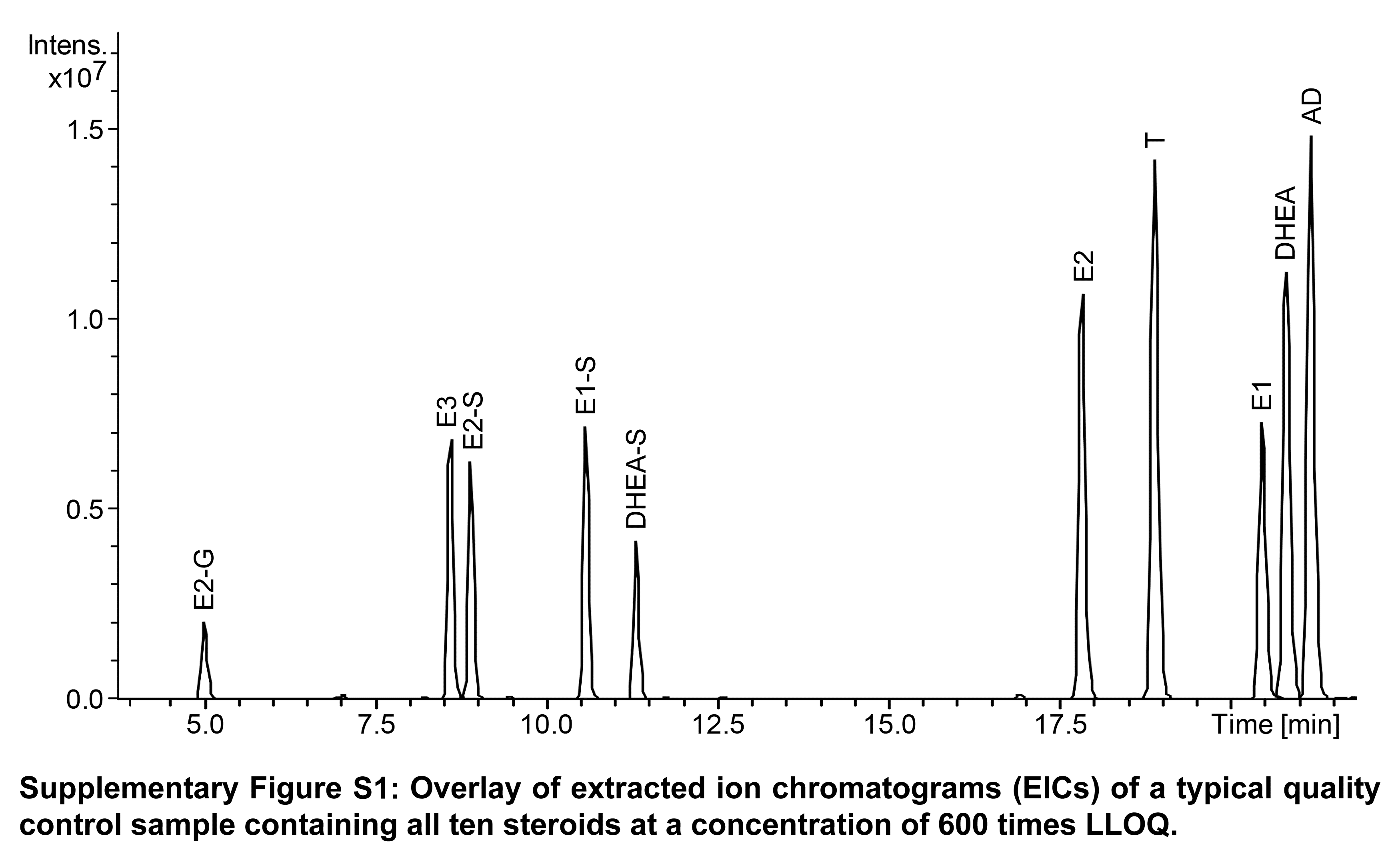

Supplement: Supplementary file 1 [file Image_1.TIF]

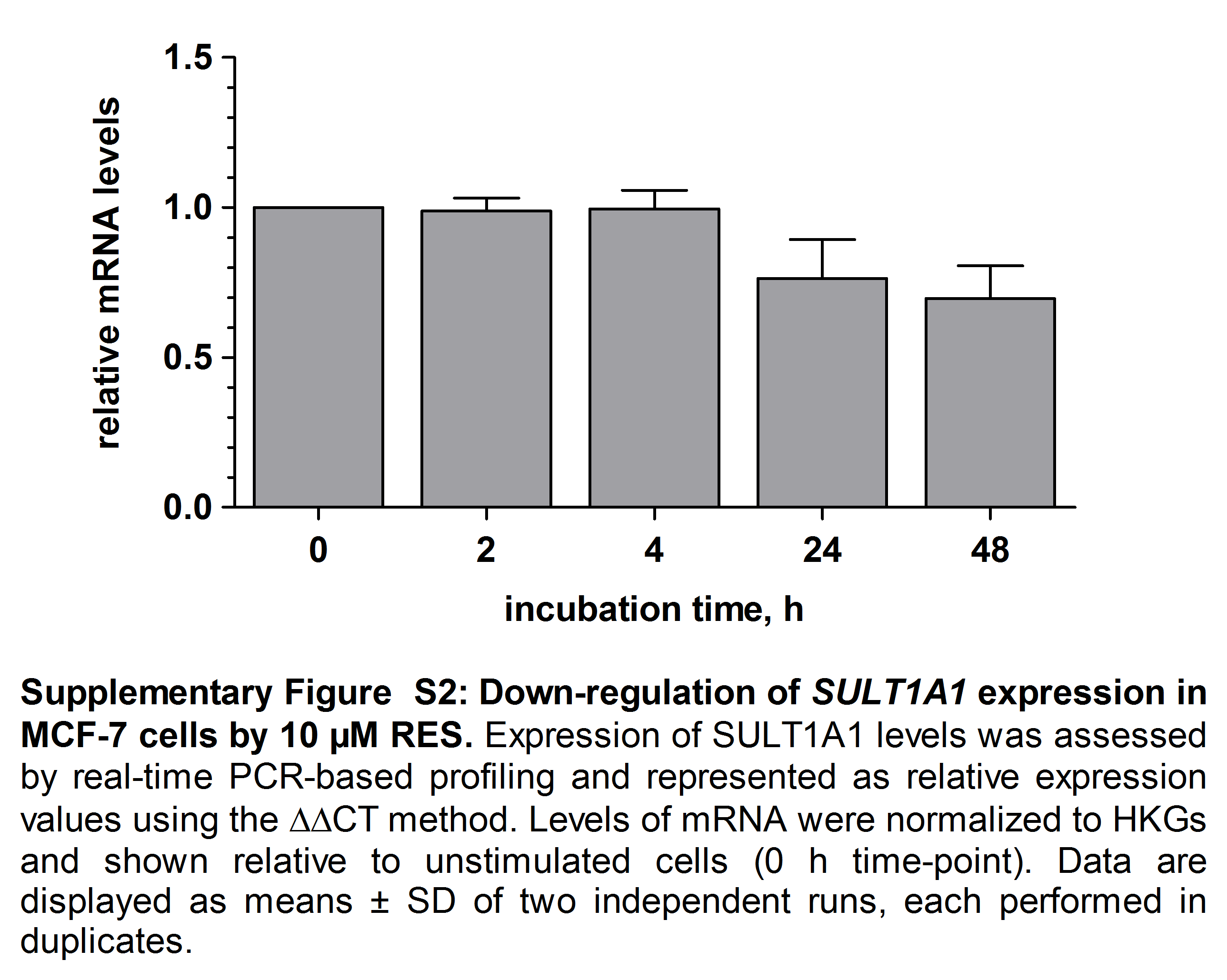

Supplement: Supplementary file 2 [file Image_2.TIF]
